# A Combined Drug Treatment That Reduces Mitochondrial Iron and Reactive Oxygen Levels Recovers Insulin Secretion in NAF-1-Deficient Pancreatic Cells

**DOI:** 10.3390/antiox10081160

**Published:** 2021-07-21

**Authors:** Ola Karmi, Yang-Sung Sohn, Henri-Baptiste Marjault, Tal Israeli, Gil Leibowitz, Konstantinos Ioannidis, Yaakov Nahmias, Ron Mittler, Ioav Z. Cabantchik, Rachel Nechushtai

**Affiliations:** 1 The Alexander Silberman Institute of Life Science, The Hebrew University of Jerusalem, Edmond J. Safra Campus at Givat Ram, Jerusalem 91904, Israel; ola.karmi@mail.huji.ac.il (O.K.); snjoshep@gmail.com (Y.-S.S.); henri.margault@mail.huji.ac.il (H.-B.M.); konstant.ioannidis@mail.huji.ac.il (K.I.); ynahmias.huji@gmail.com (Y.N.); 2 School of Medicine, The Hebrew University of Jerusalem, Jerusalem 9112102, Israel; tal.israeli@mail.huji.ac.il (T.I.); GLEIB@hadassah.org.il (G.L.); 3 Endocrinology and Metabolism Service, Hadassah Medical Center, Jerusalem 9112102, Israel; 4 Alexander Grass Center for Bioengineering, The Hebrew University of Jerusalem, Edmond J. Safra Campus at Givat Ram, Jerusalem 91904, Israel; 5 Department of Surgery, University of Missouri School of Medicine, Columbia, MO 65201, USA

**Keywords:** NAF-1 (*CISD2*), oxidative stress, iron hemostasis, Wolfram syndrome type 2 (WFS-T2), ferroptosis, insulin secretion

## Abstract

Decreased insulin secretion, associated with pancreatic β-cell failure, plays a critical role in many human diseases including diabetes, obesity, and cancer. While numerous studies linked β-cell failure with enhanced levels of reactive oxygen species (ROS), the development of diabetes associated with hereditary conditions that result in iron overload, e.g., hemochromatosis, Friedreich’s ataxia, and Wolfram syndrome type 2 (WFS-T2; a mutation in *CISD2*, encoding the [2Fe-2S] protein NAF-1), underscores an additional link between iron metabolism and β-cell failure. Here, using NAF-1-repressed INS-1E pancreatic cells, we observed that NAF-1 repression inhibited insulin secretion, as well as impaired mitochondrial and ER structure and function. Importantly, we found that a combined treatment with the cell permeant iron chelator deferiprone and the glutathione precursor N-acetyl cysteine promoted the structural repair of mitochondria and ER, decreased mitochondrial labile iron and ROS levels, and restored glucose-stimulated insulin secretion. Additionally, treatment with the ferroptosis inhibitor ferrostatin-1 decreased cellular ROS formation and improved cellular growth of NAF-1 repressed pancreatic cells. Our findings reveal that suppressed expression of NAF-1 is associated with the development of ferroptosis-like features in pancreatic cells, and that reducing the levels of mitochondrial iron and ROS levels could be used as a therapeutic avenue for WFS-T2 patients.

## 1. Introduction

Wolfram Syndrome Type 2 (WFS-T2) is a rare genetic disease found in several different populations worldwide [1,2,3,4,5,6,7]. Its early onset is characterized by severe insulin deficiency leading to juvenile onset of diabetes mellitus, progressive appearance of gastro-intestinal (GI) ulcers, abnormal platelet aggregation, sensorineural hearing loss, optical nerve atrophy, and psychosis [1,8,9,10,11]. A number of different studies identified the *CISD2* gene as the causative agent of WFS-T2, with several different mutations in the *CISD2* allele associated with different forms of WFS-T2 [1,2,3,4,6,7]. Most of the mutations in the *CISD2* gene, leading to WFS-T2, resulted in a complete or partial loss of the NAF-1 (Nutrient-deprivation Autophagy Factor-1) *CISD2* protein, a protein that normally resides on the endoplasmic reticulum (ER), the outer mitochondrial membrane (OMM), and the mitochondria-ER-associated membranes (MAM) [12]. Interactome studies of NAF-1, a member of the [2Fe-2S] NEET protein family [13], implicated this protein in the regulation of autophagy and apoptosis, and added support to the empirical evidence associating NAF-1 with dysfunctional iron metabolism, ROS formation, and Ca^2+^ signaling at the ER and mitochondria [13,14,15]. The association of NAF-1 function with altered iron and ROS metabolism gained further support from studies conducted in cancer cells [14,16,17,18,19,20,21,22,23,24]. These studies reveal that cancer cells accumulate high levels of NAF-1, and that disrupting NAF-1 function, for example by a point mutation in the NAF-1 [2Fe-2S] cluster-binding domain, or by suppressing NAF-1 protein expression using shRNA, results in enhanced levels of mitochondrial labile iron and ROS levels that trigger apoptosis of cancer cells [14,16,18,22,24]. 

The studies described above suggest a tight association between NAF-1 protein level and/or function and the protection of cells from overaccumulation of iron and ROS in their mitochondria (a form of ferroptosis, as was shown for cells with reduced expression of mitoNEET, a different member of the NEET protein family; [25,26]). Based on this potential relationship, we hypothesized that treatments that can reduce the levels of iron and ROS in the mitochondria of WFS-T2 patients could ameliorate some of the cell dysfunctions (biochemical and physiological) associated with NAF-1 deficiency in these patients. To begin addressing our hypothesis, we established an insulinoma INS-1E pancreatic β-cell model of NAF-1 stable repression (by gene Knock Down; KD; referred to here as NAF-1(−)), whereby structural and functional properties resulting from NAF-1 gene repression are demonstrably reversed by overexpressing the normal gene. We then assessed to what extent the elevated mitochondrial labile iron (mLI) and ROS (mROS) observed in NAF-1(−) insulinoma cells contributed to impaired stimulated insulin secretion, and whether alleviating mLI and mROS accumulation will improve glucose-stimulated insulin secretion. Here, we show that suppressed expression of NAF-1 in INS-1E pancreatic β-cells results in the appearance of ferroptosis-like features that include enhanced lipid peroxidation, enhanced mROS and mLI levels, shrunken mitochondria, decreased expression of glutathione peroxidase 4 (GPX4), and enhanced expression of transferrin receptor (TfR). We further show that an anti-ferroptosis treatment based on the combined action of the membrane permeant iron chelator deferiprone (DFP; [27,28]) and the glutathione precursor N-acetyl cysteine (NAC; [29,30,31]), significantly ameliorated mitochondrial labile iron and ROS levels and mitochondrial and ER structural abnormalities, as well as repairing insulin secretion of NAF-1 repressed cells. In addition, we found that treatment with the ferroptosis inhibitor ferrostatin-1 [25,32,33] decreased cellular ROS formation and improved cellular growth of NAF-1(−) INS-1E pancreatic cells. Our findings suggest that targeting ferroptosis of pancreatic cells could be used as a clinical strategy in the treatment of WFS-T2 patients. 

## 2. Materials and Methods

### 2.1. Cell Growth and NAF-1 Expression 

INS-1E β-cells were grown as previously described [11]. Plasmids containing shRNA in pGFP-RS vector (OriGene Technologies, Inc., Rockville, MD, USA) were used for repressing NAF-1 expression, whereas plasmids containing the pEGFP-N1 vector (Clontech Laboratories, Inc. Mountain View, CA, USA) were used for over-expressing NAF-1. Cell growth and transfection were performed as described earlier [16,18]. Three independent stable transfected lines were generated for each construct and transfection and used as different biological controls. 

### 2.2. Protein Blots

For protein blot analyses, cells were grown to full confluence, washed twice with 1X PBS and immediately scraped off the plate into a microcentrifuge tube with 1X Laemmli sample buffer and heated to 95 °C for 10 min. Protein gels were loaded with equal amounts of proteins and analyzed using antibodies against NAF-1 [11,16], Anti-TXNIP (Cell signaling technology, D5F3E), Anti-Thioredoxin 2 antibody (Abcam, [EPR15225] ab185544), Anti-actin (Sigma, MAB1501), Anti-IRE1 antibody (Abcam, ab37073), Anti-IRE1 (phospho S724) antibody (Abcam, [EPR5253] ab124945), anti-Mitofusin 2 antibody (Abcam, [6A8] ab56889), Anti-Glutathione Peroxidase 4 (GPX4) antibody (R&D Systems, Biotechne, MAB5457), and Anti-Transferrin Receptor Antibody (Abcam, ab84036). Goat Anti-Rabbit IgG, H&L Chain Specific Peroxidase Conjugate (Sigma, 401315) and Peroxidase-conjugated AffiniPure Goat anti-mouse IgG (H+L) (Jack-son ImmunoResearch Laboratories, AB_10015289) were used as secondary antibodies [7,24].

### 2.3. Lipid Peroxidation

Malondialdehyde (MDA) was measured using QuantiChrome TBARS Assay Kit (DTBA-100, BioAssay Systems, Hayward, CA, USA) according to the manufacturer’s instructions. Briefly, cells (5 × 10^6^) were harvested, homogenized in ice cold PBS buffer, and disrupted by sonication. Cell lysates were incubated with ice-cold 10% trichloroacetic acid and centrifuged for 5 min at 14,000 rpm. After the neutralization, the clear sample supernatant was mixed with thiobarbituric acid (TBA) solution and incubated at 100 °C for 60 min. Fluorometric assay was used for quantitative determination of lipid peroxides (thiobarbituric acid reactive substances, TBARS); this was evaluated using the Perkin Elmer EnVision 2104-0020 Multilabel Plate Reader at Ex/Em 530/550 nm. 

### 2.4. Fluorescence Probes 

Cells were cultured and imaged by epi-fluorescent microscopy for their mitochondrial labile iron (mLI) with the fluorescent probe RPA (rhodamine B-[(1,10-phenanthrolin-5-yl) aminocarbonyl] benzyl ester) (Squarix biotechnology, ME043.1, Marl, Germany), as described in [16]. Mitochondrial ROS accumulation was determined using mitoSOX^TM^ Red (Invitrogen^TM^, M36008, Massachusetts, USA), according to [18,34]. For protein carbonylation measurements, CH (Coumarin Hydrazine) (Cayman Chemical, 113707-87-2, Michigan, USA) was added at 20 µM, and fluorescence images were acquired and analyzed (excitation 365 nm, emission 430–550 nm) [35]. For mitochondrial membrane potential (MMP), we used TMRE (tetramethylrhodamine ethyl ester) (Sigma, 87917, Missouri, USA) at a concentration of 0.1 μM. Fura Red^TM^, AM, cell permeant (Invitrogen^TM^, F3020) was used to measure cytosolic Ca^2+^ by exposing cells to 4 µM for 40 min at 37 °C, in the presence of 0.02% Pluronic^®^ F-127 (Sigma, P2443). Images were analyzed with Volocity (Quorum Technologies Inc. Puslinch, ON, Canada) and/or with Image-J. Quantification of fluorescence was performed using 30 different fields (5 cells per field). Quantification of mito-SOX^TM^ fluorescence changes was performed by analyzing 9 different fields (5 cells per field) and averaged for 3 independent experiments. For all fluorescence imaging studies, different cells were plated onto microscope slides glued to perforated 3 cm diameter tissue culture plates, as previously described [16,34]. For Glutathione levels detection, ThiolTracker™ Violet (glutathione detection reagent) (Invitrogen^TM^, T10095) was used at 20 μM for 30 min at 37 °C, then measured using an Olympus FV3000 confocal laser-scanning. For cell viability, cells were seeded in 96-well plates in triplicates at a density of 2000 cells/well. PrestoBlue™ Cell Viability Reagent (Invitrogen^TM^, A13261) was used to determine cell viability on days 1, 4, and 7 with or without the addition of ferrostatin-1. Fluorescence was measured on a plate reader after 2 h of incubation at 37 °C (excitation, 560 nm, emission, 590 nm). For cell ROS measurements, CellROX™ Deep Red Reagent detection assay (Invitrogen^TM^, C10422) was used in conjunction with a Biotek plate reader (excitation 640 nm, emission 665 nm). For Mitochondrial-ER contact fluorescence imaging, cells were transfected with pDsRed2-ER plasmid to give the ER fluorescence signal, then cells were treated with the mitochondrial tracker Rhodamine 800 (Sigma, 83701). Nuclei were stained with bisBenzimide H 33,342 trihydrochloride (Sigma, 14533). Cells were then evaluated by the Olympus FV3000 confocal laser-scanning microscope, and all images were analyzed with image J.

### 2.5. Treatment of Cells with Ferrostatin-1, the Iron Chelator (DFP), and the Glutathione Precursor CGP (NAC)

Cells were incubated with or without ferrostatin-1 (3-Amino-4-cyclohexylaminobenzoic acid ethyl ester) (Sigma, Product No. SML0583), using a concentration of 2 µM, for the entire experiment period. Cells were also incubated with or without DFP (3-Hydroxy-1,2-dimethyl-4(1H)-pyridone) (Sigma, 379409). The proper concentration was optimized for each cell type and controls; DFP was added at 100 μM for 2 h to cells for mLI and mROS quantifications [11,16]. Cells were treated with/without the cellular glutathione precursor (CGP) (NAC) (*N*-Acetyl-l-Cysteine) (Sigma, Product No. A7250-10G) antioxidant. Pretreatment was optimized to 100 μM for 2 h, for the experiments of mLI and mROS levels [36,37,38]. In the experiments of insulin secretion and TEM, the DFP concentration used was 50 μM and the NAC concentration used was 100 μM, overnight.

### 2.6. Mitochondrial Bioenergetics, Oxygen Consumption Rate (OCR), Cellular Glycolysis, and Extracellular Acidification Rate (ECAR)

OCR and ECAR were measured using a Seahorse XFp, Agilent, cell mito-stress analyzer (Agilent Technologies, Inc., Santa Clara, CA, USA) with the XF Cell Mito Stress Kit and XF Glycolysis Stress Kit (Agilent Technologies, Inc., Santa Clara, CA, USA), according to the manufacturer’s instructions [39]. INS-1E β-cells (30,000) were grown to approximately 80% confluence in complete medium, 48 h before experiment. The initial medium was then exchanged with a seahorse-running medium consisting of Dulbecco’s Modified Eagle’s Medium (DMEM) base without glucose, L-Glutamine 2 mM, sodium pyruvate 1 mM, with glucose added to a final concentration of 25 mM and pH adjusted to be 7.3–7.4. Then, microplates containing cells were incubated at 37 °C without CO_2_ for 1 h before the assay. Plates were then placed into the XFp analyzer. The OCR was calculated after the sequential additions of oligomycin A 1 µM, FCCP 10 µM, antimycin A/Rotenone 0.5 µM, using an XF Cell Mito Stress Test kit. The ECAR medium initially did not contain glucose, and was then measured after the addition of glucose 25 mM. The results were expressed as mean ± SD of three independent experiments. All measurements were recorded at set-interval time points. All materials and compounds were obtained from Seahorse Bioscience [16]. Calculations of the OCR and the ECAR test parameters were performed according to the manufacturer recommendations, using the equations described in [39]. 

### 2.7. Insulin Secretion Measurement

Cells were plated in 24-well plates 48 h before insulin stimulation with high glucose concentration, as described in [11]. Cells were pre-incubated with a low glucose containing a medium of 1.7 mM after which basal insulin concentration was measure. Then, stimulation was determined after pre-incubation with high glucose (16.7 mM), as described in [11]. Insulin secretion was measured by the insulin ELISA kit (Mercodia, Uppsala, Sweden, 10-1250-01), then calculated as fold from the basal insulin concentration.

### 2.8. Mitochondria and ER Structure

Cells were grown on 8-well Permanox chamber slides and, upon reaching approximately 80% confluence, were fixed in 2.5% glutaraldehyde and 2% paraformaldehyde in 0.1 M cacodylate buffer (pH 7.4) for 2 h at room temperature, then prepared for Transmission Electron Microscope (TEM) imaging, as described in detail in [16]. The results were evaluated from 5–10 different cells randomly selected, averaged over 10–20 fields per cell; at least 100–200 mitochondria and 200–300 ER were counted. Mitochondrial and ER damages were expressed as the ratio of damaged organelle to total number of the organelle, in three independent experiments [16]. Mitochondrial length was similarly determined in three different biological repeats [16].

### 2.9. Statistics

Statistical significance tests (Student’s *t*-test) for protein expression, insulin measurements, and analysis of epi-fluorescent microscope and TEM images were performed using GraphPad Prism 8.3.1 software. The results are presented as box-and-whisker plots and include all measured data points; the line inside the box represents the median, the box represents the interquartile range, and the whiskers represent the range. Differences were statistically significant if the Student’s *t*-test produced a probability value of less than 5% (* *p* < 0.05; ** *p* < 0.01; *** *p* < 0.001).

## 3. Results

### 3.1. NAF-1-Suppressed Insulinoma Cells as a Model for Pancreatic β-Cells of WFS-T2 

**Figure 1:**
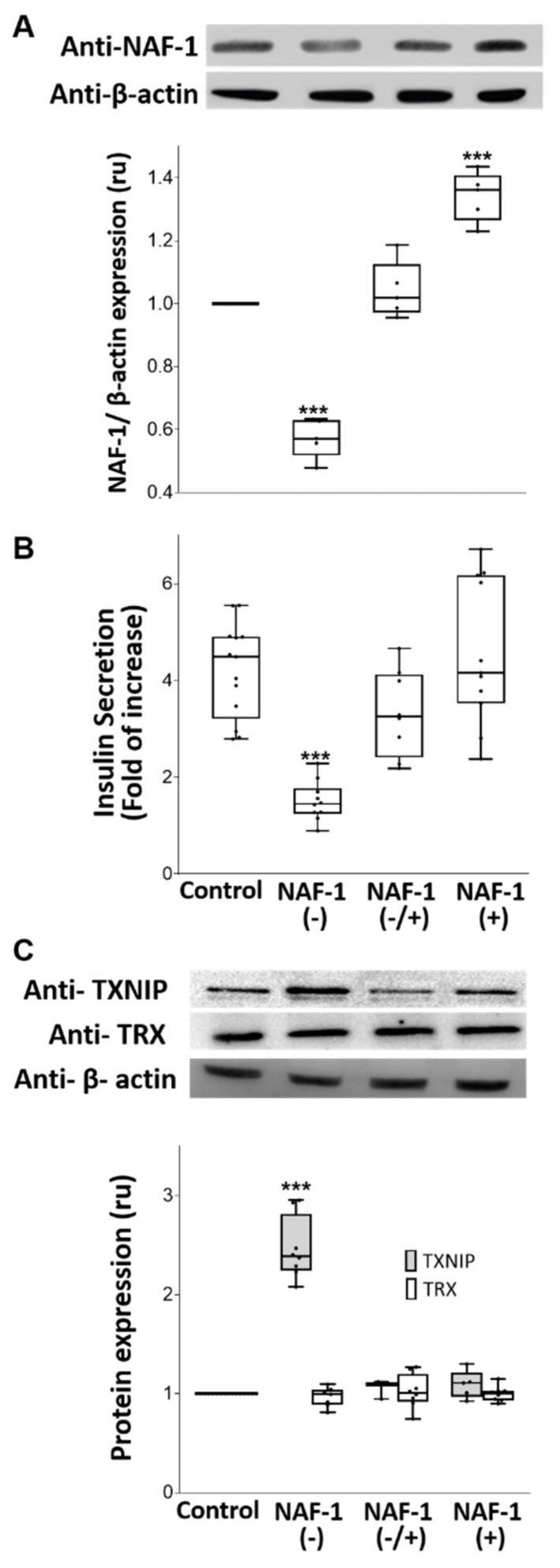
NAF-1 protein levels, insulin secretion, and TXNIP expression in INS-1E cells with repressed or overexpressed NAF-1 levels. (**A**) Protein blots (Top) and β-actin-normalized expression graph (Bottom) showing NAF-1 protein levels in control, NAF-1(−), NAF-1(−/+), and NAF-1(+) cells. (**B**) Fold change in glucose-stimulated insulin secretion of INS-1E cells expressing different levels of NAF-1. (**C**) Protein blots (Top) and β-actin-normalized expression graph (Bottom) showing TXNIP and TRX protein levels in INS-1E cells expressing different levels of NAF-1. The results are presented as box-and-whisker plots and include all data points measured in three different experiments. *** *p* < 0.001, compared to control; Student’s *t*-test, N = 3. Abbreviations: TRX, Thioredoxin; TXNIP, Thioredoxin-interacting protein.

We selected the murine insulinoma INS-1E cells for assessing the consequences of NAF-1 deficiency on pancreatic β-cells of WFS-T2 primarily on the basis that these cells respond to glucose stimuli by secreting insulin. Following NAF-1 shRNA transfection, we selected 3 independent stable clones of INS-1E cells expressing ~50% lower levels of the NAF-1 protein compared with WT control (Figure 1A). Importantly, these NAF-1(−) cells showed a commensurately lower glucose-stimulated insulin secretion ability compared with normal cells (Figure 1B) and a 2.5-fold increase in the level of the thioredoxin-interacting protein (TXNIP) (but not thioredoxin (TRX)) (Figure 1C). TXNIP and TRX play antagonistic roles in β-cell function and diabetes pathophysiology, and accumulation of TXNIP in insulinoma cells is typically a sign of oxidative stress [40,41,42]. To ascertain that the changes in cell functions are attributable to a reduction in NAF-1 per se, control WT and NAF-1(−) cells were further transfected with WT NAF-1, referred to here as NAF-1(+) and NAF-1(−/+), respectively (3 independent stable clones for each vector were generated). As shown in Figure 1, the repressed expression of NAF-1, as well as glucose-stimulated insulin secretion and the enhanced expression of TXNIP in NAF-1(−) INS-1E cells could be significantly reversed by transfection of NAF-1(−) cells with the WT NAF-1 gene. 

### 3.2. Mitochondrial, ER, and MAM Structural Changes in NAF-1(−) Cells

**Figure 2:**
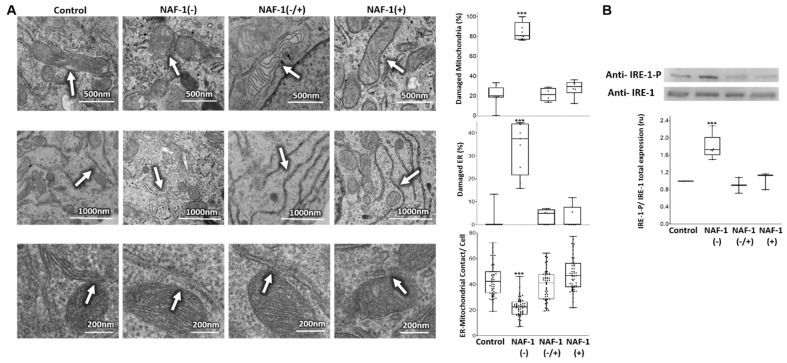
Mitochondrial and ER structural alterations and phosphorylation of IRE-1 in INS-1E cells with repressed or overexpressed NAF-1 levels. (**A**) Representative TEM images (left panels) and statistical analysis (right graphs) of alterations in mitochondrial (top), ER (middle), and ER-mitochondrial contact points (bottom) structures in control, NAF-1(−), NAF-1(−/+), and NAF-1(+) cells. (**B**) Protein blots (top) and graph (bottom) showing the ratio between IRE-1 and phosphorylated IRE-1 (IRE-1P) in INS-1E cells expressing different levels of NAF-1. The results are presented as box-and-whisker plots and include all data points measured in three different experiments. *** *p* < 0.001, compared to control; Student’s *t*-test, N = 600 for mitochondria and mitochondrial-ER contact and N = 900 for ER. Abbreviations: IRE, serine/threonine-protein kinase/endoribonuclease.

**Figure 3:**
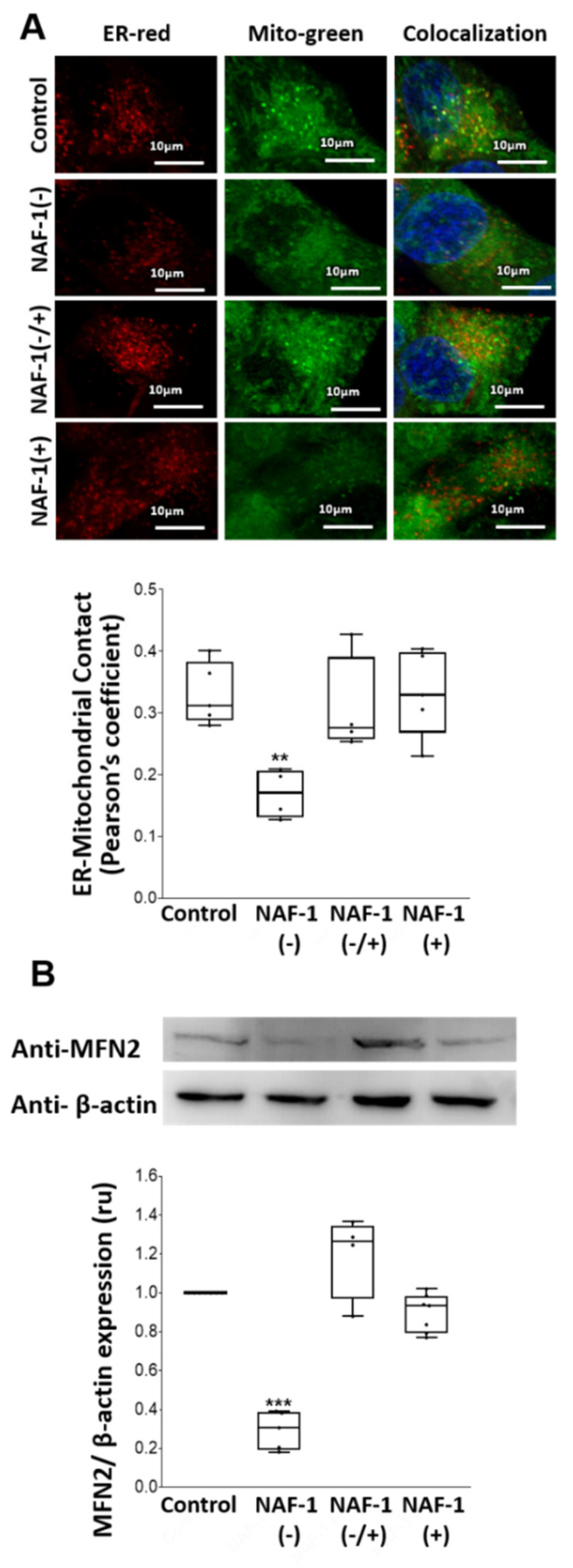
Quantification of ER-mitochondrial contacts using confocal microscopy and expression of MFN2 in INS-1E cells with repressed or overexpressed NAF-1 levels. (**A**) Representative confocal images of INS-1E control, NAF-1(−), NAF-1(+), and NAF-1(−/+) cells co-expressing an ER-, mitochondria- and nucleus-targeted red, green, and blue fluorescent proteins, respectively (top). Yellow (green-red merged fluorescence) denotes contact points between the ER and the mitochondria. Quantification graphs showing the number of ER–mitochondrial contact points per cell (bottom). (**B**) Protein blots (top) and β-actin-normalized expression graph (bottom) showing MFN2 protein levels in INS-1E cells expressing different levels of NAF-1. The results are presented as box-and-whisker plots and include all data points measured in three different experiments. ** p < 0.01, *** p < 0.001, compared to control; Student’s *t*-test, N = 50. Abbreviations: MFN2, mitofusin 2.

NAF-1(−) cells displayed various abnormalities in mitochondria size, cristae integrity, and ER structure (Figure 2A), possibly as a result of ER stress, as suggested by the increased expression of phosphorylated IRE-1 expression (Figure 2B), an important component of the unfolded protein response (UPR). Moreover, the number of membranal contacts between mitochondria and ER (MAMs) decreased, suggesting fewer interactions between mitochondria and the ER through these structures (Figure 2A and Figure 3A). This reduced tethering was further supported by measuring the levels of mitofusin 2 (MFN2; [43,44,45]), a protein implicated in the maintenance of MAM integrity (Figure 3B). Importantly, mitochondrial, ER, and MAM abnormalities found in NAF-1(−) cells were largely corrected upon re-expression of WT NAF-1 in NAF-1(−) cells (Figure 2 and Figure 3).

### 3.3. Biochemical and Physiological Changes in NAF-1(−) Cells 

**Figure 4:**
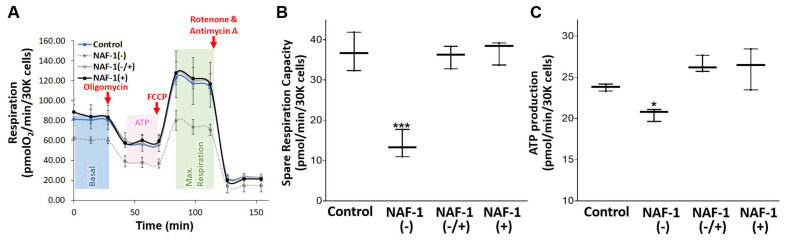
Mitochondrial respiration and ATP production in INS-1E cells with repressed or overexpressed NAF-1 levels. (**A**) Seahorse-generated plots of mitochondrial respiration obtained for the different lines. Data are presented in normalized values. (**B**) Spare respiratory capacity calculated from (**A**). (**C**) ATP levels calculated from (**A**). Results in (**A**) were normalized to the number of cells used (30,000). Data in (**B**,**C**) are presented as box-and-whisker plots and include all data points measured in three different experiments. * p < 0.05, *** p < 0.001, compared to control; Student’s *t*-test, N = 3.

**Figure 5:**
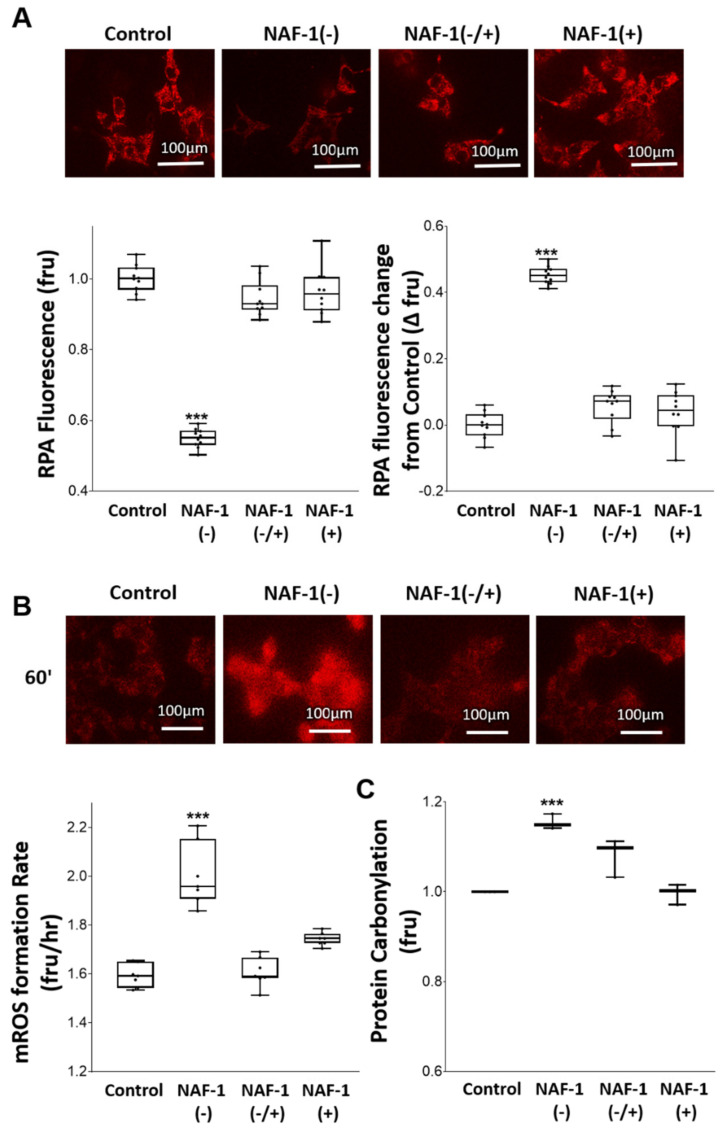
Mitochondrial labile iron (mLI), ROS (mROS), and protein carbonylation in INS-1E cells with repressed or overexpressed NAF-1 levels. (**A**) Representative epi-fluorescent images of mitochondrial RPA fluorescence (top), and quantification of mitochondrial RPA fluorescence (bottom graphs) in INS-1E cells with repressed or overexpressed NAF-1 level (left graph is for total RPA fluorescence and right graph is for change in RPA fluorescence from control). Quenching of RPA fluorescence indicates mitochondrial labile iron accumulation. (**B**) Representative epi-fluorescent images of mitochondrial mito-SOX^TM^ fluorescence (top), and quantitative analysis (bottom, left), of mitochondrial mito-SOX^TM^ fluorescence (indicating mitochondrial superoxide accumulation), in INS-1E cells with repressed or overexpressed NAF-1 level. (**C**) Cellular content of carbonylated proteins in the different INS-1E cell lines with repressed or overexpressed NAF-1 levels, measured with coumarin hydrazine (CH). The results are presented as box-and-whisker plots and include all data points measured from three different experiments. *** p < 0.001, compared to control; Student’s *t*-test, N = 450 for (**A**), N = 150 for (**B**), and N = 450 for (**C**).

To determine whether the structural changes observed in NAF-1(−) INS-1E (Figure 2 and Figure 3) were accompanied by biochemical and physiological changes in mitochondrial function, we measured mitochondrial respiration, ATP production, mLI, and mROS in NAF-1(−) cells. A significant reduction (~40%) in mitochondrial functions of NAF-1 repressed cells was observed in mitochondrial maximal respiratory capacity, an indicator of functional mitochondrial mass (Figure 4A), cell respiration (Figure 4B), and ATP production (Figure 4C); all indicative of depleted mitochondrial reserves. Restoration of NAF-1 expression to wildtype levels commensurately restored these affected functions. By contrast, extracellular acidification rate (ECAR) of NAF-1(−) did not significantly change, suggesting that, at least in INS-1E cells, a deficiency in NAF-1 levels does not impact glycolysis (Figure S1). In this respect, insulinoma cells are different from epithelial breast cancer cells in which glycolysis was activated upon NAF-1 protein suppression [34]. In addition to its effect on mitochondrial respiration (Figure 4), the reduced expression of NAF-1 led to an increase in mitochondrial labile iron (mLI) (Figure 5A), a rise in the levels of mitochondrial ROS (mROS) (Figure 5B), and an increase in protein carbonylation levels (Figure 5C). Similar to our findings described above for mitochondrial structure and function (Figure 2, Figure 3 and Figure 4), restoration of NAF-1 expression to wildtype levels commensurately restored mLI, mROS, and protein carbonylation levels to near WT levels (Figure 5). Additional changes that were restored by recovering the expression of NAF-1 in NAF-1(−) cells were changes in mitochondrial membrane potential (MMP; Figure S2), and cytosolic Ca^2+^ levels (Figure S3). 

### 3.4. Pharmacological Amelioration of NAF-1 Depletion in INS-1E Cells 

**Figure 6:**
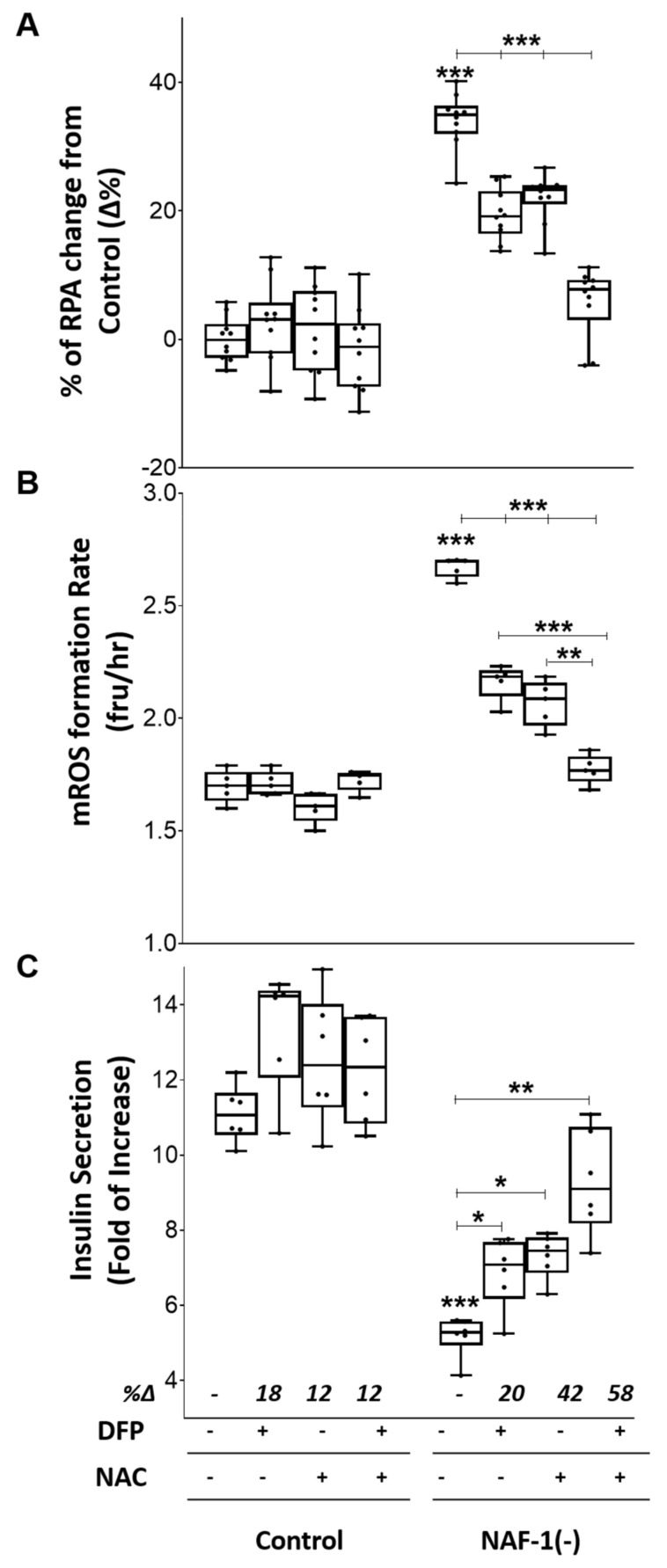
Pharmacological amelioration of enhanced mLI and mROS, and decrease insulin production in NAF-1(−) INS-1E cells with a combined treatment of DFP and NAC. (**A**) Mitochondrial labile iron levels in control and NAF-1(−) cells in the presence or absence of DFP (50 µM) and/or NAC (100 µM). (**B**) Same as in (**A**), but for mROS levels. (**C**) Fold change in glucose-stimulated insulin secretion of control and NAF-1(−) cells in the presence or absence of DFP and/or NAC. The results are shown as box-and-whisker plots and include all data points measured from three different experiments. * p < 0.05, ** p < 0.01, *** p < 0.001, compared to control; Student’s *t*-test, N = 450 for (**A**), N = 150 for (**B**), and N = 3 for (**C**). Abbreviations: DFP, deferiprone; NAC, N - acetyl cysteine.

**Figure 7:**
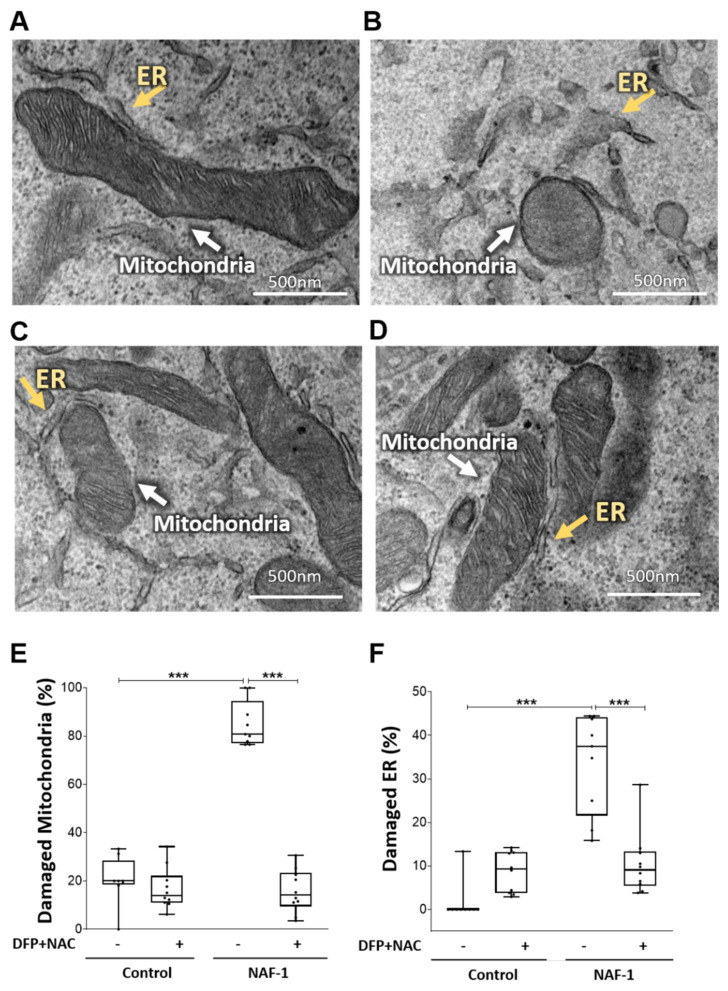
Pharmacological amelioration of mitochondrial and ER morphology in NAF-1(−) INS-1E cells with a combined DFP and NAC treatment. (**A–D**) Representative TEM images of control and NAF-1(−) INS-1E cells treated or untreated with a NAC (100 µM) + DFP (50 µM) combination (A, Control. (**B**) NAF-1(−). (**C**) Control treated with DFP+ NAC. (**D**) NAF-1(−) treated with DFP+ NAC). Yellow and white arrows indicate ER and mitochondria, respectively. (**E**,**F**) Statistical analysis of mitochondrial (**E**), or ER (**F**), damage in NAF-1(−) INS-1E cells following the combined DFP+ NAC treatment. The results are shown as box-and-whisker plots and include all data points measured from three different experiments. *** p < 0.001, compared to control; Student’s *t*-test, N = 200 for mitochondria, N = 300 for ER. Abbreviations: DFP, deferiprone; NAC, N-acetyl cysteine.

The findings described above reveal that a reduction in NAF-1 protein level in INS-1E insulinoma cells results in a significant decrease in glucose-stimulated insulin secretion (Figure 1), which was accompanied by structural changes to mitochondria and ER (Figure 2 and Figure 3), as well as a decrease in mitochondrial functions (Figure 4 and Figure 5). Most importantly among these are the enhanced accumulation of mLI and mROS, which have been directly linked to NAF-1 function in cells [11,16,18,34]. Because an increase in mLI can lead to an increase in mROS, which would in turn impact mitochondrial function and structure [14], we tested whether treatments such as chelation of mLI and/or inhibition of mROS accumulation could ameliorate insulin secretion and mitochondrial and ER morphology of INS-1E NAF-1(−) cells. To test the potential impact of such treatments on pancreatic pathophysiology, we followed mLI, mROS, and stimulated insulin secretion in pancreatic NAF-1(−) cells treated with the membrane permeant iron chelator deferiprone (DFP; [27,28]) and the antioxidant glutathione precursor N-acetyl cysteine (NAC; [29,30,31]), separately and in combination. As shown in Figure 6A,B, treatment with DFP and/or NAC ameliorated the NAF-1(−) induced increase in mLI and mROS, with the combined application of these two agents having the most significant effect. Although each of these agents significantly improved insulin secretion, their combined action was additively corrective (Figure 6C). In addition to correcting some of the biochemical effects of NAF-1 deficiency (Figure 6), the combined treatment of INS-1E NAF-1(−) cells with DFP+NAC ameliorated the impact of NAF-1 deficiency on mitochondrial and ER morphology (Figure 7). Treatments of INS-1E NAF-1(−) cells with DFP and/or NAC, capable of suppressing ferroptosis [46,47,48], were therefore able to correct some of the biochemical, morphological, and functional (insulin secretion) phenotypes of NAF-1 deficiency (Figure 6 and Figure 7).

**Figure 8:**
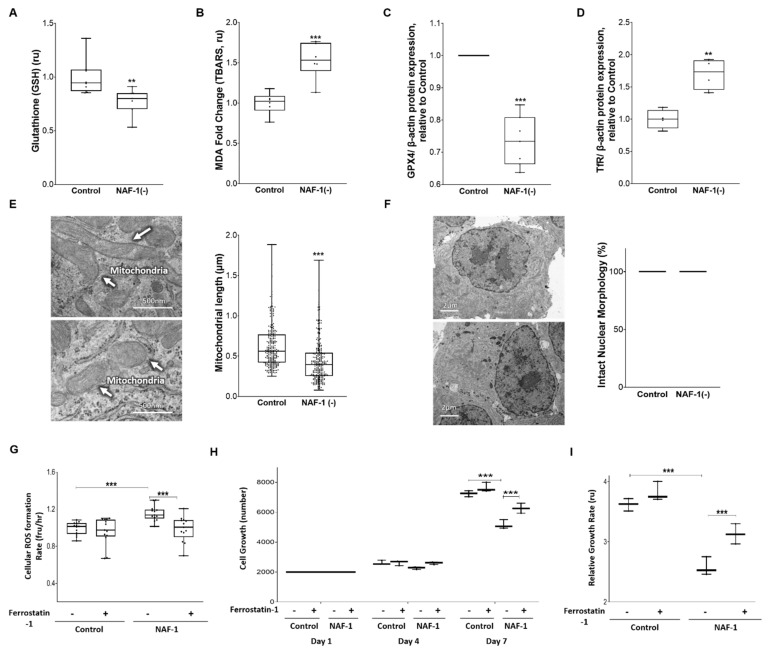
Presence of ferroptosis-like symptoms in NAF-1(−) pancreatic cells. Reduced glutathione (GSH) levels (**A**), increased lipid peroxidation (**B**), decreased GPX4 expression (**C**), enhanced TfR expression (**D**), and shortened mitochondria (**E**) (representative image: top, control; bottom, NAF-1-deficient) in control and NAF-1(−) pancreatic cells. (**F**) Morphology of nuclei in control and NAF-1(−) cells (representative image: top, control; bottom, NAF-1-deficient). (**G**) Cellular ROS levels in control and NAF-1(−) cells in the presence or absence of 2 µM, ferrostatin-1. (**H**,**I**) cell growth of control and NAF-1(−) cells (measured by counting cell numbers (**H**) or using PrestoBlue^TM^ (**I**)) in the presence or absence 2 µM ferrostatin-1. The results are shown as box-and-whisker plots and include all data points measured from three different experiments. ** *p* < 0.01, *** *p* < 0.001, compared to control; Student’s *t*-test, N = 3 for (**A**,**B**,**G**,**H**,**I**), N = 5 for (**C**,**D**), N = 250 for (**E**), N = 10 for (**F**). Abbreviations: GSH, reduced glutathione; MDA, malondialdehyde; GPX4, glutathione peroxidase 4; TfR, transferrin receptor.

The rise in mLI, mROS, and cytosolic Ca^2+^ levels, upon NAF-1 depletion (Figure 5, Figure S3), and the finding that this rise could be suppressed by treatment with NAC and/or DFP (Figure 6), could suggest that NAF-1 deficiency in insulinoma cells triggers ferroptosis [49,50]. To test this possibility, we measured the total levels of reduced glutathione (GSH), the levels of lipid peroxidation, the expression of GPX4 and TfR, and the length of mitochondria in control and NAF-1(−) INS-1E cells. In addition, we studied the effect of the ferroptosis inhibitor ferrostatin-1 [25,32,33] on these cells. Compared with wildtype, NAF-1(−) cells had lower GSH levels (Figure 8A), enhanced lipid peroxidation (Figure 8B), suppressed expression of GPX4 (Figure 8C), enhanced expression of TfR (Figure 8D), and shortened mitochondria (Figure 8E). By contrast, no apoptosis features such as nuclear degradation were observed in NAF-1(−) cells (Figure 8F). In addition, ferrostatin-1 treatment of INS-1E NAF-1(−) cells resulted in a significant reduction in cellular ROS formation and improved cell growth (Figure 8G). The results presented in Figure 5, Figure 6, Figure 7 and Figure 8 suggest therefore that NAF-1 deficiency causes the enhanced accumulation of mLI and mROS and that this accumulation triggers ferroptosis-like features of INS-1E cells.

## 4. Discussion

NAF-1 repression or deficiency in animal and cell models results in cellular dyshomeostasis of calcium, [Fe-S] protein functions, iron status, and ROS production [4,16,34,51], as well as in the activation of cell death processes such as apoptosis [12,13,14,34]. To what extent the changes in cell properties imparted by the experimental repression of NAF-1 fully recapitulate the impaired properties of different tissues found in WFS-T2 patients is still under thorough evaluation. Here, we used the pancreatic INS-1E cellular model system to study how NAF-1 repression impairs insulin secretion in pancreatic cells (Figure 1), recapitulating a key feature of the WFS-T2 phenotype. The repression of NAF-1 induced changes in mitochondrial, ER, and MAM morphology and affected multiple mitochondrial properties (Figure 2, Figure 3, Figure 4 and Figure 5). First and foremost, we ascertained that it is the actual reduction in NAF-1 per se and not the transfection that led to the changes in the affected cellular properties. This was accomplished by re-expressing wild type NAF-1 in the NAF-1 repressed β-pancreatic INS-1E cellular model and showing significant restoration of properties affected in mitochondria, ER, and MAM structure-function. These included mitochondrial and ER morphology, mitochondrial labile iron and ROS accumulation, as well as changes in calcium levels, respiration, and insulin secretion (Figure 1, Figure 2, Figure 3, Figure 4 and Figure 5). Changes in mitochondrial morphology and insulin secretion have previously been shown to result from ROS-induced MMP damage [52], ER stress [53], and an unfolded protein response that can lead to cell death [54]. As MAM and cell Ca^2+^ have also been implicated in glucose-induced insulin secretion [55], NAF-1 presumed role in regulating subcellular Ca^+2^ concentrations could take place via putative interactions with the inositol 1,4,5-triphosphate receptor (IP3R) involved in Ca^2+^ release from the ER [56] and/or sarco/endoplasmic reticulum Ca^2+^-ATPase (SERCA2) proteins that control Ca^2+^ fluxes into the ER [57,58,59]. 

Aside from NAF-1(−) induced changes in mitochondria-ER MAM integrity (Figure 2 and Figure 3), the multiple changes observed in INS-1E cells upon NAF-1 suppression recapitulate what we and others have observed in previous studies with non-endocrine cells in culture and with animal models of WFS-T2 (i.e., knockouts of NAF-1) [15,60,61,62]. The novel finding of this work is therefore the establishment of a causative association between NAF-1 repression that leads to a mitochondrial labile iron rise and an increased mROS formation that triggers ferroptosis, and the impairment of β-cell insulin secretion. This association was experimentally demonstrated by the combined application of the mitochondrial iron chelator DFP and the GSH precursor NAC (Figure 6 and Figure 7), as well as by the application of the ferroptosis inhibitor ferrostatin-1 [63] (Figure 8). First, we show that reduced GSH levels were significantly decreased in NAF-1(−) cells, possibly explaining the increased mROS formation that could also be attenuated by DFP+NAC, a combined treatment known to reduce these two arms of ferroptosis [49,50]. We further show that suppressed NAF-1 expression results in enhanced lipid peroxidation, suppressed expression of GPX4, enhanced expression of TfR, and the appearance of smaller mitochondria (Figure 8), all classic hallmarks of ferroptosis [64]. GSH may also have a role in iron homeostasis in addition to its antioxidant redox buffer effects [65,66]. Moreover, the combined DFP and NAC treatment of pancreatic INS-1E NAF-1(−) cells led to a significant amelioration of mLI and mROS, as well as enhanced insulin secretion and improved mitochondria and ER morphologies (Figure 6 and Figure 7), which are some of the hallmarks of WFS-T2 [11,60]. Although the use of low levels of DFP was previously shown to cause enhanced accumulation of ROS in cancer cells [67,68], the concentrations used in our study were much higher. These concentrations were previously shown to also suppress ROS formation in cancer cells [16,69] and are acceptable for the application of DFP in other systems [70,71]. Pending additional studies conducted using different cell lines, as well as different model organisms, our findings could provide a novel rationale for pharmacological intervention for symptomatic improvement in an otherwise incurable disease.

## 5. Conclusions

Currently, it is clinically impractical to change the whole-body intracellular expression levels of NAF-1 in vivo in WFS-T2 patients. Here, we succeeded in alleviating the abnormalities caused by NAF-1 reduction using a pharmacological approach (DFP+NAC). The combined DFP and NAC treatment of pancreatic INS-1E NAF-1(−) cells repaired to near-normal levels the abnormalities in mLI and mROS accumulation, mitochondrial and ER morphologies, and insulin secretion. These findings suggested that ferroptosis could be playing an important role in WFS-T2 β-pancreatic pathophysiology, supported by the correcting effect of ferrostatin-1 on cellular growth as well as cellular ROS generation of INS-1E NAF-1(−) cells. Our findings suggest therefore that targeting ferroptosis of pancreatic cells, for example, by the combined pharmacological application of the clinically approved DFP and NAC, could be used as a viable strategy in the treatment of WFS-T2 patients and perhaps patients suffering from other metabolic diseases that result in enhanced mLI and mROS levels [72,73,74,75,76]. 

## Data Availability

Data is contained within the article and Supplementary Materials.

## Supplementary Materials

The following are available online at https://www.mdpi.com/article/10.3390/antiox10081160/s1.

## Abbreviations

CH	Coumarin hydrazine
DFP	Deferiprone
ECAR	Extracellular acidification rate
ER	Endoplasmic reticulum
GI	Gastro-intestinal
GPX4	Glutathione peroxidase 4
GSH	Glutathione
IP3R	Inositol 1,4,5-triphosphate receptor
IRE-1	Serine/threonine-protein kinase/endoribonuclease IRE1
KD	Knock down
MAM	Mitochondria-ER-associated membranes
MDA	Malondialdehyde
MFN2	Mitofusin 2
mLI	Mitochondrial labile iron
MMP	Mitochondrial membrane potential
mROS	Mitochondrial reactive oxygen species
NAC	N-acetyl cysteine
NAF-1	Nutrient-deprivation autophagy factor-1
OCR	Oxygen consumption rate
OMM	Outer mitochondrial membrane
ROS	Reactive oxygen species
RPA	Rhodamine B-[(1,10-phenanthrolin-5-yl) aminocarbonyl] benzyl ester
SERCA2	Sarco/endoplasmic reticulum Ca2+-ATPase
TBARS	Thiobarbituric acid reactive substances
TEM	Transmission electron microscope
TfR	Transferrin receptor
TMRE	Tetramethylrhodamine ethyl ester
TRX	Thioredoxin
TXNIP	Thioredoxin-interacting protein
UPR	Unfolded protein response
WFS-T2	Wolfram syndrome type 2
WT	Wildtype
