# Supplementary material for: A Combined Drug Treatment That Reduces Mitochondrial Iron and Reactive Oxygen Levels Recovers Insulin Secretion in NAF-1-Deficient Pancreatic Cells
Source: Antioxidants (Basel). 2021 Jul 21;10(8):1160. doi: 10.3390/antiox10081160 (PMC8388971; doi:10.3390/antiox10081160)
Supplement: Supplementary file 1 [file antioxidants-10-01160-s001.zip › antioxidants-1269067-supplementary.pdf]

Supplementary Materials For:

Article

A Combined Drug Treatment That Reduces Mitochondrial Iron and Reactive Oxygen Levels Recovers Insulin Secretion in NAF-1-Deficient Pancreatic Cells

Ola Karmi [1], Yang-Sung Sohn [1], Henri-Baptiste Marjault [1], Tal Israeli [2], Gil Leibowitz [2,3], Konstantinos Ioannidis [1,4], Yaakov Nahmias [1,4], Ron Mittler [5,*], Ioav Z. Cabantchik [1,*] and Rachel Nechushtai [1,*]

[1] The Alexander Silberman Institute of Life Science, The Hebrew University of Jerusalem, Edmond J. Safra Campus at Givat Ram, Jerusalem 91904, Israel; ola.karmi@mail.huji.ac.il (O.K.); snjoshep@gmail.com (Y.-S.S.); henri.margault@mail.huji.ac.il (H.-B.M.); konstant.ioannidis@mail.huji.ac.il (K.I.); ynahmias.huji@gmail.com (Y.N.)

[2] School of Medicine, The Hebrew University of Jerusalem, Jerusalem, 9112102, Israel; tal.israeli@mail.huji.ac.il (T.I.); GLEIB@hadassah.org.il (G.L.)

[3] Endocrinology and Metabolism Service, Hadassah Medical Center, Jerusalem, 9112102, Israel

[4] Alexander Grass Center for Bioengineering, The Hebrew University of Jerusalem, Edmond J. Safra Campus at Givat Ram, Jerusalem 91904, Israel

[5] Department of Surgery, University of Missouri School of Medicine, Columbia, MO 65201, USA

* Correspondence: mittlerr@missouri.edu (R.M.); ioav.caban@mail.huji.ac.il (I.Z.C.); rachel@mail.huji.ac.il (R.N.)

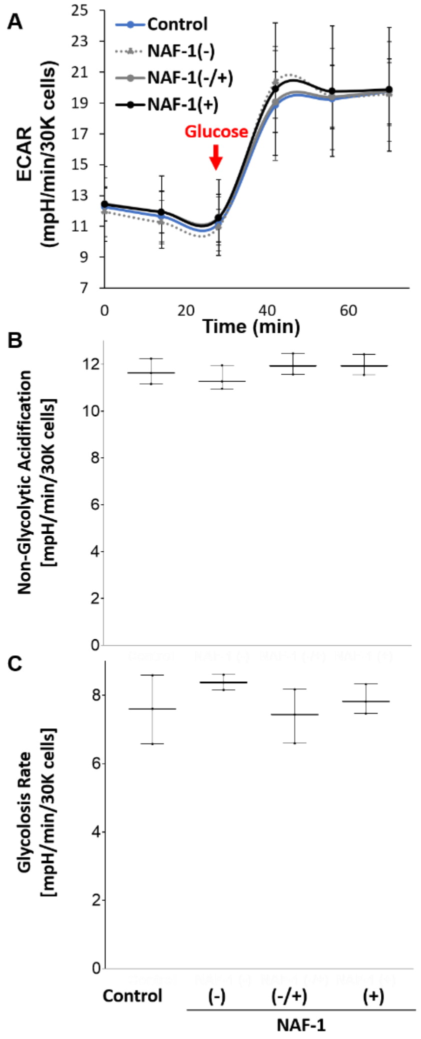

Supplementary Figure S1. Extracellular acidification rate (ECAR) in control and NAF-1 repressed cells. (**A**) Seahorse-generated plots of ECAR obtained for the different lines. Data are presented in normalized values. (**B**) Non-glycolytic basal rate in the different NAF-1 expressing INS-1E cells, calculated from (A). (**C**) Glycolysis rates in the different INS-1E cells calculated from (A). The ECAR results are shown as box-and-whisker plots and include all data points measured from three different experiments, normalized to the number of cells used per experiment (30,000 cells). Abbreviations: ECAR, extracellular acidification rate.

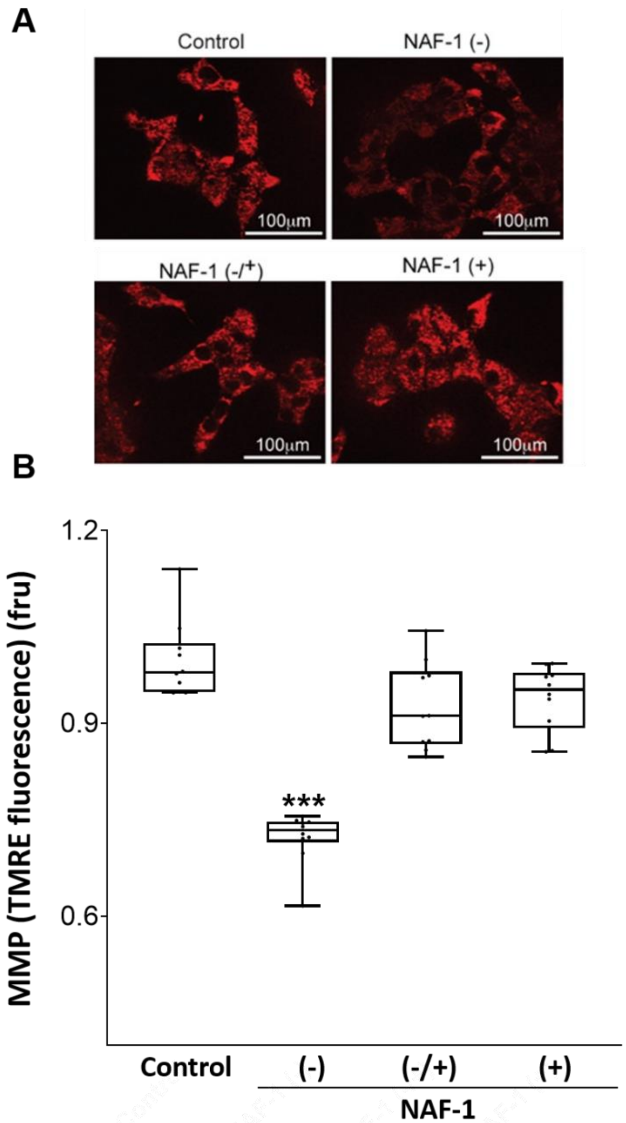

Supplementary Figure S2. Mitochondrial membrane potential (MMP) in control and NAF-1 repressed cells. (**A**) Epi- fluorescence images of cells labeled with the MMP probe TMRE. The cell fluorescence is mostly in mitochondria, where the probe accumulates potentiometrically. (**B**) Quantitative analysis of TMRE fluorescence levels of cells expressing different levels of NAF-1. The results are shown as box-and-whisker plots and include all data points measured from three different experiments. ******$P<0.001$*, compared to control; Student's t-test, N = 150.

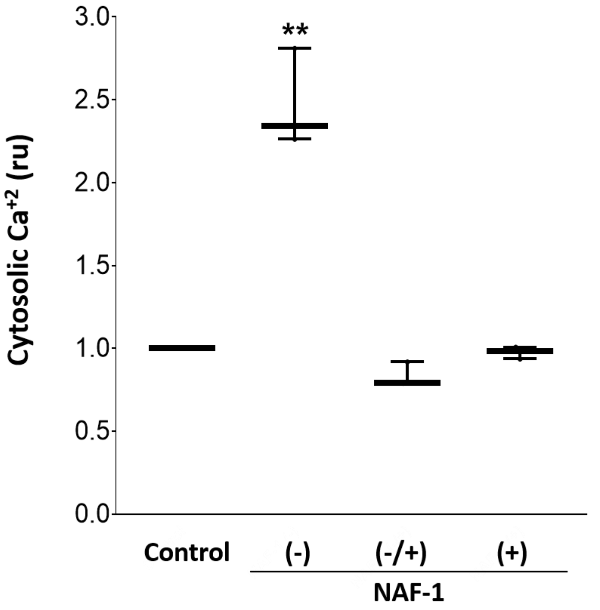

Supplementary Figure S3. Cytosolic Ca^{2+} levels in control and NAF-1 repressed cells. Semi-quantitative measurements of cytosolic Ca^{2+} levels were performed with Fura Red™ preloaded as Fura Red™-AM. Fluorescent microscopy was used to detect the fluorescent signal, by analyzing 5 cells per field, in total 150 cells from 30 fields. The results are shown as box-and-whisker plots and include all data points measured from three different experiments. $^{**}P<0.01$, $^{***}P<0.001$, compared to control; Student's t-test.
